# Does a Recycling Carbon Tax with Technological Progress in Clean Electricity Drive the Green Economy?

**DOI:** 10.3390/ijerph19031708

**Published:** 2022-02-02

**Authors:** Weijiang Liu, Min Liu, Tingting Liu, Yangyang Li, Yizhe Hao

**Affiliations:** 1 Center for Quantitative Economics, Jilin University, Changchun 130012, China; liuwj@jlu.edu.cn; 2 Business School, Jilin University, Changchun 130012, China; liutingting19@mails.jlu.edu.cn (T.L.); liyangyang20@mails.jlu.edu.cn (Y.L.); haoyz20@mails.jlu.edu.cn (Y.H.)

**Keywords:** carbon tax recycling policy, green economy, technological progress, CGE model, triple dividend, carbon emissions

## Abstract

The environmental issue is a significant challenge that China faces in leading the development of the green economy. In this context, reducing CO_2_ emissions is the key to combatting this problem. Taking the 2017 social accounting matrix (SAM) as the database and combing macroeconomic parameters from previous studies, this article constructed the environmentally computable general equilibrium (CGE) model as an analytical model to analyze the economic–environmental–energy impacts of recycling carbon tax with technological progress in clean electricity. We found that when the rate of clean electricity technological progress reaches 10%, the carbon recycling tax that reduces corporate income taxes will achieve a triple dividend of the carbon tax, namely, promoting economic development, reducing carbon emissions, and improving social welfare. In the meantime, on the basis of carbon tax policies that raise the price of fossil energy, clean electricity technological progress will help accelerate the transformation of electricity structure, reduce the proportion of thermal power generation, and better promote emission reduction. In addition, due to the high carbon emission coefficient, coal contributes significantly to carbon emission reduction. Therefore, China should implement a carbon tax recycling policy supplemented by the progress of clean power technology as soon as possible to better promote green economy development.

## 1. Introduction

Green and sustainable development is a core driver of economic development worldwide. However, the growing amount of carbon emissions poses a tremendous threat to it. The current status of China’s carbon emissions has remained underwhelming. In 2020, China’s primary energy demand increased by 2.1%, and total carbon dioxide emissions increased by 0.6%. By contrast, the proportion increased. Specifically, fossil energy consumption accounted for 85.2% of total energy consumption. The high proportion of fossil energy has led to higher CO_2_ emissions. As the largest carbon emission producer in the world, China is actively committed to adopting more effective policies and measures to reduce carbon emissions [1]. On 22 September 2020, President Xi Jinping proposed at the general debate of the 75th United Nations General Assembly that “China will increase its nationally determined contributions (NDC), striving to reach the peak of carbon dioxide emissions by 2030, further, try to achieve carbon neutrality by 2060”. Later, at the Climate Ambition Summit, he announced that China’s carbon dioxide emissions per unit of GDP will drop by more than 65% compared to 2005 by 2030, the proportion of non-fossil energy to primary energy consumption will reach about 25%, and wind and solar power generation capacity will reach more than 1.2 billion kilowatts. In response, it is necessary to study how China can promote carbon reduction, achieve carbon emission commitments, and drive the development of a green economy.

In the process of promoting the development of a green economy, the power industry, as an industry with a relatively large volume of emissions, should be of particular concern. Coal-fired power generation is the main source of power generation in most parts of the world [2]. As for China, in 2020, nuclear power generation accounted for 4.7% of produced power; hydropower generation accounted for 17%; and renewable energy power generation accounted for 11%. Based on the above data, clean powers’ proportions are relatively low. Coal-fired power generation was still the primary source of power generation, accounting for 63% of the power produced (https://www.bp.com/, (accessed on 23 January 2022)). China’s coal reserves are relatively sufficient, and coal-fired power generation has the advantages of controllability and stability. Therefore, thermal power generation relies primarily on coal combustion [3]. It is undeniable that high carbon emissions accompany the combustion process. According to the IEA, the global electricity and heat sector contributes 42% to CO_2_ emissions, with China contributing a larger share in comparison (https://www.iea.org/, (accessed on 23 January 2022)). At the same time, in the light of the CEADs database statistics, the industries involved in the production and supply of electricity, steam, and hot water produce the highest carbon dioxide emissions (https://www.ceads.net/, (accessed on 23 January 2022)). China’s power industry has enormous potential for emission reduction. The green development and transformation of China’s fuel power generation industry have become a general trend.

Countries worldwide have been exploring corresponding measures to achieve green economic development, including carbon pricing policies, which can advance progress towards the achievement of climate goals [4]. Carbon emission reduction incentives based on carbon pricing policies help the competitive market play a role, reducing carbon emissions further. Specifically, carbon tax and trading policies are two effective carbon pricing policies [5,6]. The carbon tax policy is a price control strategy levied on fossil energy’s carbon content or emissions, whereas the carbon trading policy is a total quantity control strategy [7,8]. In addition, the carbon tax is simpler and faster than the Emission Trading Scheme (ETS) for developing countries within the short to medium term [9]. At the same time, a more rational carbon tax corrected the intra-sector distortions created by ETS [10]. However, the operation of a carbon tax policy causes the price of related energy to rise, affecting corporate investment and household consumption, which is not conducive to economic growth or the improvement of social welfare. In this regard, a properly designed carbon tax recycling policy is the key to achieving the double dividend (promoting economic growth and reducing carbon emissions) [11]. To be more specific, a carbon tax recovery policy refers to the imposition of a carbon tax while returning it to residents or businesses in various forms, such as reducing existing distortionary tax rates (lower corporate income tax, resident income tax, and others), affecting socio-economic variables such as corporate income and resident income [12]. Furthermore, as crucial factors of production, the efficiency and technology of energies are also effective measures that can be used to avoid the adverse effects of carbon taxes. Technological progress can curb carbon emission intensity while not reducing long-term economic growth [13]. Combined with the above analysis, both carbon tax recovery and technological progress are effective and important tools to maintain economic growth under carbon reduction targets.

Nevertheless, existing studies mainly focus on individual research on carbon tax policy and the impact of technological progress on carbon emission reduction, whereas there are few studies on the combination of carbon tax recycling policy and technological progress. Moreover, the analysis does not focus on technological progress in the clean electricity sector, applying the same rate of technological progress to all sectors and making it difficult to distinguish the impact of technological progress on specific industries. In this regard, this article constructs an environmental CGE model that includes the subdivision of power sectors, combining the carbon tax recycling policy with the progress of clean electricity technology for research, intending to explore the carbon tax’s emission reduction and economic growth effect. Furthermore, we explore the possible carbon tax policy’s social welfare effects on this basis. Our study is conducive to realizing the recovery of the green economy and achieving China’s Nationally Determined Contribution (NDC), thus promoting China’s ecological civilization construction and green economy development.

The structure of this article is as follows: Section 2 compares relevant literature, Section 3 describes the model construction and data source, and Section 4 analyzes the results of the policy combination simulation. The last section summarizes the conclusions drawn by the model and puts forward relatively reasonable policy recommendations to provide relevant policy references for national policymakers.

## 2. Literature Review

### 2.1. Research on Electricity and Carbon Emissions

Since the Industrial Revolution, the rapid increase in greenhouse gas emissions has caused severe environmental and health problems [14]. To date, the issue of climate change caused by a large amount of greenhouse gas emissions is still receiving widespread attention from countries all over the world. Among them, CO_2_ is a kind of greenhouse gas that has attracted much attention. Focusing on the power industry that emits more carbon dioxide, many scholars have studied the relationship between electricity and carbon dioxide emissions. The consumption of electricity has a detrimental effect on carbon emissions. In particular, electricity generated from fossil fuels causes damage to carbon emissions and is the primary source of carbon emission reduction. Renewable power generation can weaken the adverse impact of power generation on carbon emissions [15,16]. Combining the STIRPAT model and the panel threshold model, Lin and Li found that electricity usage affects carbon emissions negatively, especially when clean electricity accounts for a relatively high proportion of the energy used [17]. Wong and Zhang conducted a natural experiment on Australia’s carbon pricing mechanism (CPM), finding that the carbon tax has little impact on areas where renewable energy power generation accounts for a relatively high proportion of the energy produced. After abolishing the carbon tax, the market behavior, which had begun to shift from coal-fired power generation to other energy sources, was reversed, showing that coal-fired power has higher carbon emissions [18]. Yang and Song introduced clean coal-fired power generation to study the effects of emission reductions in the coal-fired power generation sector [19]. Haxhimusa and Liebensteiner evaluated the impact of electricity demand reductions on emission reductions based on the analysis of COVID-19 shocks to electricity demand using an applied econometric model in an instrumental variables framework [20]. Fang et al. analyzed the characteristics of carbon emissions and carbon intensity in the power sector from a spatial perspective using the Moran index. Additionally, based on the multi-regional input-output table, inter-provincial embodied carbon transfer in the power sector was explored [21]. By using a system dynamics approach, Mostafaei et al. discovered a new model for using renewable electricity to reduce CO_2_ emissions [22]. The empirical results of the above literature show that electricity impacts carbon emissions. Therefore, it is important that we dig deeper into the issue of carbon emission reduction within the power sector as a starting point.

### 2.2. Research on Carbon Tax and Carbon Tax Recycling Policy

For a long time, the carbon tax policy has been a policy tool used to reduce greenhouse gas emissions and weaken the negative impact of climate change. In 2020, the carbon tax policy took effect or was in the process of being implemented in 30 countries and regions [23]. Many scholars have made corresponding assessments on the effect of the carbon tax policy’s implementation, mainly reflected in three aspects. First of all, the existing literature has tested the emission reduction effect of the carbon tax and proved the effectiveness of carbon tax for carbon emission reduction. Secondly, as for the macroeconomic impact of carbon taxes, Yamazaki’s research found that although income-neutral carbon taxes do not harm employment, carbon taxes have caused employment to shift from carbon-intensive industries to cleaning service industries [24]. Furthermore, carbon taxes affect the GDP negatively and affect economic growth [25,26]. Finally, regarding the effects of the carbon tax on residents’ welfare, Khastar et al. demonstrated that the Finnish carbon tax had a specific negative impact on social welfare [27].

There is a need to recycle carbon taxes in the face of the negative impact of carbon taxes on the economy and social welfare. Pearce showed that the carbon tax policy has a “double dividend” effect. Specifically, for one thing, a carbon tax can affect carbon emissions and improve environmental quality; for another thing, a carbon tax can offset corporate income tax to increase corporates’ investment and enhance the efficiency of economic operations. Implementing appropriate carbon tax recovery policies can effectively increase residents’ and enterprises’ income, further stimulate consumption and investment, and promote economic growth [28]. The realization of the double dividend depends on the recycling method of the carbon tax revenue. Specifically, there are two types of carbon tax recovery: the income-neutral principle and the income-positive principle [29]. The principle of income neutrality introduces carbon taxes while canceling or reducing some taxes to maintain income neutrality [30], while under the principle of income positivity, the government recycles the additional carbon tax received back to the household or corporate sectors to increase the corresponding income. Ojha et al. used a recursive dynamic CGE model to study the issue of carbon tax recycling in India. They considered that based on carbon emission reduction, the recycling of carbon tax would benefit economic growth and weaken the inequality of income distribution [31]. Li et al. simulated the impact of a carbon tax on employment under various carbon tax recovery scenarios [12]. Generally speaking, recycling carbon taxes reduces the tax burden and the negative impact on the economy, and different carbon tax recovery schemes will have different results [32]. The ultimate acceptance and successful implementation of a carbon tax depend on how the carbon tax revenue is utilized [33]. Most scholars study whether the carbon tax’s double dividend will be realized. At the same time, there are few considerations of social welfare, causing the absence of rational exploration and discovery of the carbon tax’s triple dividend.

### 2.3. Research on Technological Progress and Carbon Emission Reduction

Carbon tax recycling policy has indeed contributed to carbon emission reduction. At present, achieving emission reduction targets while ensuring sustainable economic development is an important challenge for the government [34]. Technological progress is undoubtedly the most promising solution to the challenge [13]. Chen et al. found that technological advances reduced carbon emissions during the study period in China [35]. Considering that technological progress is an essential factor in promoting carbon emission reduction, Wu et al. introduced technological progress into the CGE model to predict the carbon emission situation of the global economy and evaluate the effect of different technological advancements on carbon emission reduction [36]. After simulation and prediction analysis, Guo et al. once again posited that the continuous improvement of total factor productivity is one of the critical conditions that needs to be met for China to achieve the goal of energy regulation [37]. Using the best technologies to reduce unnecessary energy consumption and improve energy efficiency deserves attention [38]. However, the relationship between technological progress and carbon dioxide emissions is still complex and requires in-depth research [39]. Wang et al. comprehensively studied the relationship between technological progress and CO_2_ emissions and found heterogeneity in the effects of technological progress on different economic sectors and emission agents [40]. As far as technological progress in the power industry is concerned, there is still room for growth. For example, the intermittent and random nature of renewable energy power generation has brought massive challenges to the operation and planning of the power system [41]. Sofia et al. approved that the electricity market must be reformed to encourage the development of zero-emission technologies [42]. Nonetheless, few documents have targeted their analysis on the effects of technological progress in the clean power sector, lacking a reasonable expectation of technological advancement in a specific clean power sector.

### 2.4. Research on CGE Model Involving Electricity

The literature mentioned above uses various theories and empirical models for research. Nevertheless, the CGE model is widely used in policy simulation. As a tool for policy simulation analysis, CGE can study issues such as carbon taxes, carbon trading rights, and energy efficiency improvements. In addition, many scholars have studied power-related issues using the CGE model. Based on the static CGE model, He et al. analyzed the impact of coal price adjustments on the power industry and electricity price adjustments on China’s macroeconomics [43]. Meng constructed a CGE model to examine the impact of Australia’s carbon tax policy on the power sector [44]. Lin and Jia used the improved CGE model to analyze only the relevant effect of the power industry’s carbon trading market, indicating that the annual decline factor can reach 0.5% when allocating carbon allowances in the power sector [45]. Mardones and Brevis constructed a social accounting matrix (SAMEA) with environmental accounts and found differences between power sectors. If the power sector is not highly decomposed, the simulation effects of energy and environmental policies are biased [46]. Cui et al. used CGE models with different nesting structures and power sector substitution flexibility to study reducing the impact of renewable power reduction policies on economic development and the environment. Their studies have shown that reducing renewable electricity cuts can achieve various green benefits, such as cutting CO_2_ emissions generated from the power sector and improving the real GDP [47]. Zhang et al. assessed the impact of three policies, including technological progress, on power generation, carbon emissions, and prices, analyzing how nuclear power generation could be promoted [48]. Nong built a CGE model of the electricity environment to study South Africa’s carbon tax policy [49]. Based on these works, we selected the CGE model for research.

From the above research review, most of the existing articles analyze and simulate scenarios where only one variable changes, and it is impossible to further compare the results of the combined effects of multiple strategies. Although previous studies have recognized the negative impact of clean electricity on carbon emissions, none have analyzed whether its technological progress will further promote carbon emission reduction. To our knowledge, this study is the only of its kind attempting to fill this gap in understanding. Motivated by these limitations, this article mainly put forth the following contributions. First, this article simulated a combination of a carbon tax recycling policy and clean power technology, exploring the effectiveness of this interaction; second, this article introduces technological progress of the clean electricity sector and divides the power sector in detail. In this way, we analyzed the critical role of the combination of technological progress in the clean power sector and a carbon tax recycling policy in a targeted manner to identify the heterogeneity of different power sectors; finally, under the principle of tax neutrality, based on the double-dividend theory of carbon tax, this article conducts further excavations to explore carbon tax’s triple-dividend effect.

## 3. Methodology and Data

### 3.1. CGE Model Structure

**Figure 1:**
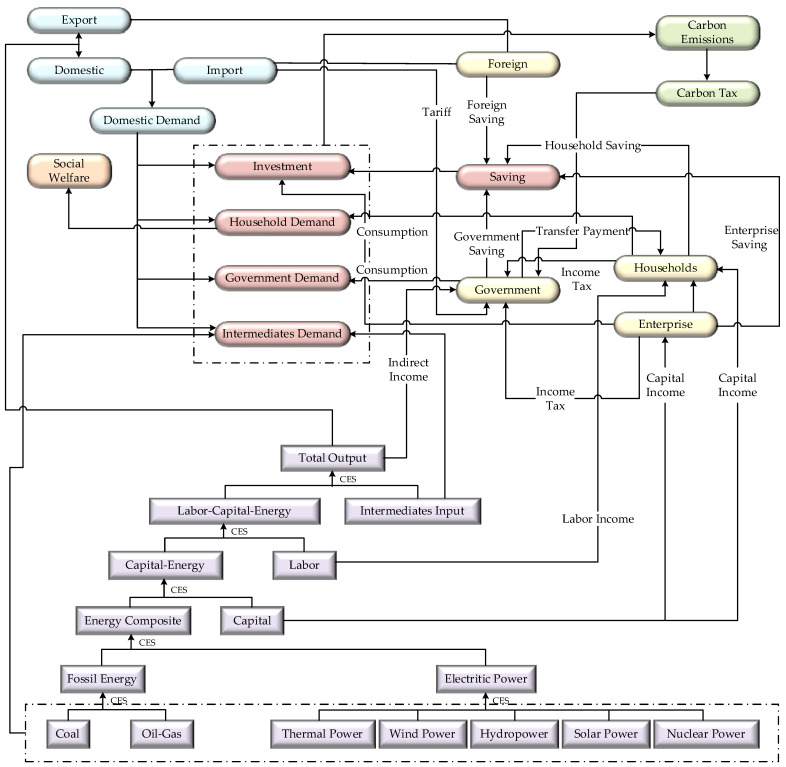
The framework of the CGE model.

The CGE model builds on the Walras general equilibrium theory and portrays the real economic system. Precisely, the model consists of a production module, a trading module (domestic product demand and distribution), an institutional module, an equilibrium module, a social welfare module, and a carbon emission and carbon tax module, covering three types of institutions, namely the resident, the corporate, and the government. Figure 1 presents a general framework of the model.

#### 3.1.1. Production Module

The production module of the CGE model in this paper includes five nested layers, all linked by a constant elasticity of substitution (CES) function, except for the intermediate inputs, which use the Leontief function. In the first layer, fossil energy is subdivided into coal and oil–natural gas, and electricity is divided into five types according to different generation technologies, namely thermal, hydro, nuclear, solar, and wind power. The second layer is the synthesis between fossil energy and electricity. The third layer is the synthesis of capital and energy complexes. The fourth layer is the synthesis between capital, energy complexes, and labor, i.e., the value-added component of the synthesis. The fifth layer synthesizes capital, energy, and labor complexes with intermediate inputs. 

#### 3.1.2. Trade Module

The allocation of domestic products in the trade module takes the form of a CET function, which describes the distribution strategy of commodities produced by domestic production activities between domestic production and domestic sales and exports. The demand for domestic products takes the form of an Armington function, which further compounds domestic production and domestic sales with imported goods to form domestic market commodities.

#### 3.1.3. Institutional Module

The institutional module mainly includes residents, enterprises, and government modules. We set the corresponding income and expenditure functions. In the resident module, residents’ income primarily comes from labor income, capital income, and transfer payments from enterprises and the government. The income of residents is used for consumption and saving, and this paper used a linear function to describe the consumption behavior of residents. In the enterprise module, enterprise income is mainly derived from capital income, and enterprise savings are derived from enterprise after-tax income minus enterprise transfers to residents. In the government module, the government income consists of indirect taxes, customs duties, residential and corporate income taxes, and carbon taxes. These revenues are in turn used for government consumption as well as savings, etc.

#### 3.1.4. Balance Module

Four balances are set up in the equilibrium module, namely factor market equilibrium, product market equilibrium, savings–investment equilibrium, and balance-of-payments equilibrium. In this paper, we adopted the neoclassical closure rule, which assumes that the prices of labor and capital are endogenous and that full employment is achieved in the labor and capital markets. Therefore, in factor markets, the aggregate supply of labor and capital is equal to the aggregate demand for that factor. For product markets, the aggregate supply of products equals the aggregate demand. For saving and investment, when the economy reaches equilibrium, both aggregate investments equal aggregate savings. For the balance of payment equilibrium, we chose the closure rule with the exchange rate as the endogenous variable and foreign savings as the exogenous variable. Meanwhile, we set up the nominal GDP and real GDP equations. Nominal GDP is defined as the sum of total capital input, total labor input, and indirect tax revenue, while real GDP consists of consumptions, investments, and net exports.

#### 3.1.5. Social Welfare Module

In the social welfare module, we introduced Hicks equivalence changes to measure the change in social welfare following a policy shock [50]. Specifically, the Hicks equivalent change calculates the change in utility before and after the policy’s implementation, using the commodity’s price before the policy shock as the standard. As shown in Equation (1):

(1) $$EV=E\left(U^{s},PQ^{b}\right)-E\left(U^{b},PQ^{b}\right)=\sum_{i}PQ_{i}^{b}\cdot HD_{i}^{s}-\sum_{i}PQ_{i}^{b}\cdot HD_{i}^{b}$$

where, EV measures the change in the welfare of the population, $E\left(U^{s},PQ^{b}\right)$ and $E\left(U^{b},PQ^{b}\right)$ denote the expenditure required to achieve population welfare $U^{s}$ and $U^{b}$, respectively, at $PQ_{i}^{b}$ consumer prices before the policy shock. $HD_{i}^{s}$ and $HD_{i}^{b}$ represent the populations’ consumption after and before implementing the policy, respectively. When EV is positive, it means that the population’s welfare improved after the policy’s implementation, and contrarily, if it is negative, the population’s welfare has been compromised.

#### 3.1.6. The Carbon Emission and Carbon Tax Module 

This module calculated CO_2_ emissions, further introducing a carbon tax. To analyze the carbon tax policy effect, we introduced a carbon tax shock module:

(2) $$CTAX_{i}=tc\cdot \sum_{j}E_{i,j}\cdot \theta_{j},$$

(3) $$CTAX_{j}=tc\cdot \sum_{i}E_{i,j}\cdot \theta_{j},$$

(4) $$TCTAX=\sum_{j}CTAX_{j},$$

(5) $$t_{cj}=\frac{CTAX_{j}}{PQ_{j}\cdot QQ_{j}},$$

where, j=coal,oil-gas. Among the above formulas, $\theta_{j}$ indicates the CO_2_ emission factor per unit of energy from fossil fuels (coal and oil–gas), tc denotes the amount of carbon tax levied per ton of CO_2_ emissions, $t_{cj}$ indicates the corresponding carbon tax rate of fossil energy source j. $CTAX_{i}$ and $CTAX_{j}$ denote the amount of carbon tax levied on the sector i and the intermediate input component of fossil energy j, respectively. TCTAX represents the total amount of carbon tax. This paper levied a carbon tax on intermediate energy inputs in the production process, not on the final demand sector.

### 3.2. Data

The data basis for the CGE model is the SAM table. The SAM table describes the supply and use flows between accounts in the System of National Accounts (SNA) and their equilibrium relationships, comprehensively portraying the economic linkages among sectors. Based on the 2017 China Input-Output tables (http://data.stats.gov.cn/, (accessed on 23 January 2022)), taxation, capital flows, and other relevant data, we constructed the SAM table in this paper. Among them, indirect taxes, as well as taxes such as income tax, were derived from the 2018 China Financial Yearbook (https://www.epsnet.com.cn/, (accessed on 23 January 2022)), and the capital and balance of payment transfer data are from the 2018 China Statistical Yearbook (http://www.stats.gov.cn/tjsj/ndsj/, (accessed on 23 January 2022)). In the SAM table, we set up six main account types: production activity accounts, commodity accounts, factor accounts, institutional accounts, investment accounts, and foreign accounts. According to the research needs, the CGE model constructed in this paper contains 13 industries and 13 commodities; the factor accounts include labor and capital, and the institutional accounts cover households, corporates, and the government. Specifically, in terms of methods of relevant research, combining relevant data from the 2019 China Electricity Yearbook (https://navi.cnki.net/knavi/yearbooks/, (accessed on 23 January 2022)) and the 2017 China Industrial Statistics Yearbook (https://data.cnki.net/Yearbook/, (accessed on 23 January 2022)), we divided the electricity sector [46,51]. Electricity production consists of five power generation technologies [37]: thermal, hydro, nuclear, wind, and solar power, including four clean power generation sectors: hydro, nuclear, wind, and solar power. Of these, coal and oil–gas only have intermediate inputs to thermal power, and there are no intermediate inputs to the clean power sector. 

Calibration and setting of parameters in the model mainly include the carbon emission factor and the elasticity of substitution factor. The CO_2_ emission factor for fossil energy is calculated based on the ratio of CO_2_ emissions to actual energy consumption, where the CO_2_ emissions data were obtained from International Energy Statistics (https://www.eia.gov/, (accessed on 23 January 2022)). As for the setting of the relevant elasticity coefficients required for the model, we refer to the relevant research literature [52,53,54], setting the production function elasticity of substitution coefficient, the CET function relevant elasticity of substitution, and the Armington function elasticity of substitution. The share parameters were calibrated using the variable base year data and the elasticity of substitution calculations.

## 4. Simulation Analyses

Carbon tax policy is an effective tool for reducing carbon emissions, but it may adversely affect economic development and social welfare. This article analyzes the economic–environmental–energy effects of carbon tax policies and carbon tax recycling policies with and without clean power technology progress under specific carbon emission reduction targets and explores the possible triple dividends of carbon taxes. According to related simulation studies and empirical analysis, we set the simulated clean power sector’s technological progress rate as 1%, 5%, and 10%, finding changing trends in various variables as technology advances [55,56]. We conducted relevant analysis against the benchmark scenario (no carbon tax and carbon tax recovery policy). The five scenarios are as follows:

Scenario 1: Assuming that the annual carbon dioxide emissions are reduced by 5% compared to the benchmark scenario, a carbon tax is levied on the intermediate energy input in the production process, while no carbon tax is levied on the final demand sector.

Scenario 2: Assuming that the annual carbon dioxide emissions are reduced by 5% compared to the benchmark scenario, a carbon tax is levied on the intermediate energy input in the production process, while no carbon tax is levied on the final demand sector. Under the premise of ensuring the neutrality of government revenue, the resident income tax rate will be reduced.

Scenario 3: Based on Scenario 2, we set different rates of technological progress for the clean power sector.

Scenario 4: Assuming that the annual carbon dioxide emissions are reduced by 5% compared to the benchmark scenario, a carbon tax is levied on the intermediate energy input in the production process, while no carbon tax is levied on the final demand sector. Unlike Scenario 2, under the premise of ensuring the neutrality of government revenue, the corporate income tax rate will be reduced.

Scenario 5: Based on Scenario 4, we set different rates of technological progress for the clean power sector.

### 4.1. The Impact of Policy Scenarios on Macroeconomic Variables

Based on the scenario shocks set in this article, the prices of input factors and relevant products were affected accordingly, and the entire economic system was further exerted through enterprise modules, resident modules, and other modules. Taking into account the three goals of economic growth, carbon emission reduction, and social welfare improvement, Table 1 presents the percentage changes in nominal GDP, real GDP, total carbon dioxide emission intensity, residential income, and enterprise income relative to the baseline scenario under different policy scenarios, showing the resulting social welfare.

#### 4.1.1. Nominal GDP and Real GDP

It is clear from Table 1 that in Scenarios 1, 2, and 4, the nominal and real GDP decline. As carbon tax implementation leads to a fall in total output, the capital required for production falls, the price of capital decreases, and capital revenues fall. The labor price is the benchmark price, which keeps labor income constant. Indirect taxes do not account for a large proportion of the GDP, so the nominal GDP declines. For the real GDP, fossil energy price also rises due to implementing the carbon tax policy. First, the rising cost of fossil energy leads to an increase in the price of related commodities and a decline in overall consumer demand; secondly, the decrease in the demand for fossil energy by various sectors causes a decrease in enterprise output and investment; finally, the domestic levy of carbon taxes increases domestic commodity-related prices. Further, net exports are reduced. In summary, the real GDP falls.

As technological advances in clean electricity continue to rise, the capital required for production increases, and capital prices and capital revenues increase, leading to a gradual trend of increasing nominal GDP under both carbon tax recovery scenarios. Regarding the actual GDP, in Scenarios 2 and 3, when the carbon tax is recycled to residents to reduce residents’ income tax, residents’ income tax is lowered, which boosts the relevant consumer demand of residents. Likewise, the carbon tax is recycled to corporates, the corporate income tax is reduced, and the corporates’ savings rise. According to the neoclassical closure rule used in this paper, investment is determined by savings. Thus, corporate investment rises further, promoting economic growth. In addition, the progress of clean power technology has further mobilized the enthusiasm of clean power companies in production, and the actual GDP continues to rise.

#### 4.1.2. Resident Income

Resident income comprises labor income, capital income, and transfer payments from enterprises and the government. The model uses labor price as the benchmark price for related simulations as far as labor income is concerned. Therefore, the labor income of residents remains unchanged. In terms of capital income, the carbon tax policy raises the price of fossil energy, especially the cost of production for resource-intensive enterprises, causing a fall in total output and a decline in the demand for capital. Correspondingly, capital prices and total capital income are reduced. Residents receive capital income according to a set proportional coefficient, and therefore, their capital income decreases accordingly. In Scenario 1, due to the implementation of the carbon tax policy, government revenues increase, which increases the transfer payments to residents in turn, so overall residents’ income can be improved. As for Scenario 2, the carbon tax is transferred to residents at a reduced income tax rate, which declines residents’ income tax. However, the government income decreases relative to Scenario 1, resulting in a decrease in government transfers to residents. In general, residents’ income decreased. In Scenario 3, with the advancement of clean power technology, the clean power output increases significantly, and the demand for capital rises, which corresponds to the rise in capital prices. The corporate capital income and transfer payments to residents increase. In addition, residents’ capital income also increases, making residents’ income gradually rise compared with Scenario 2. Similarly, in Scenario 4, the government’s transfer payments to residents decrease, and residents’ revenues decline. In Scenario 5, the progress of clean power technology causes residents’ income to gradually rise compared with Scenario 4.

#### 4.1.3. Corporate Income

The corporate income is derived from the income generated by the capital elements input. Total capital income is allocated to the business on a proportionate share base. Like the relevant analysis of residents’ income, in Scenarios 1, 2, and 4, the carbon tax levy reduces capital income and corporate income. For Scenarios 3 and 5, advances in clean power technology further increase the demand for capital in the production process. Capital prices gradually rise, capital income increases, and corporate income also shows a gradual upward trend. In the end, under the advancement of 10% clean electricity technology for both scenarios where the carbon tax is recycled to residents and businesses, business revenues rise by 0.0240% and 0.0439% relative to the baseline scenario, respectively.

#### 4.1.4. Social Welfare

As for Scenario 1, on the one hand, the increase in residents’ income leads to an increase in residents’ demand. On the other hand, the levy of carbon taxes increases the production costs of enterprises, further increasing product prices and reducing consumer demand, which ultimately causes negative social welfare. Regarding Scenario 2, the carbon tax is recycled for residents. Although the decline in capital income and government transfer payments to residents causes a decrease in residents’ income, the income tax rate of residents decreases, which eventually increases residents’ demand, and social welfare becomes positive. Regarding Scenario 3, as the rate of clean power technology progress gradually increases, capital income rises, corporate transfer payments to residents increase, residents’ consumption demand further increases, and social welfare continues to improve.

For Scenario 4, the carbon tax is recycled to corporates, residents’ income is decreased, and consumption is reduced. Compared to Scenario 1, the social welfare is smaller. For Scenario 5, the carbon tax is recycled to corporates, and as the clean electricity technology advances, corporate further increases the production in the clean electricity sector, which in turn increases the corresponding consumption of the residents. Moreover, due to the increase in firms’ income, production in other sectors also rises to various degrees, increasing the corresponding consumption. As for residents, the increase in residents’ income also drives up residents’ consumption. Therefore, social welfare continues to rise as well. Eventually, social welfare becomes positive when clean electricity technology advances by 10%.

#### 4.1.5. CO_2_ Emission Intensity

The main reason for the reduction in carbon dioxide emission intensity is that the price of fossil energy has risen, the use of fossil energy has fallen, and more clean energy sources have replaced fossil energy with higher carbon content. Accordingly, there is a corresponding reduction in CO_2_ emissions. In particular, the clean power sector technology further promotes fossil energy conversion, the share of clean power usage rises, and the industrial structure is adjusted and upgraded. Under the conditions of a certain decline in carbon dioxide, the nominal GDP has a rising trend, and the intensity of carbon dioxide emissions continues to decline. As shown in Table 1, with the progress of clean power technology, the reduction in carbon dioxide emission intensity gradually increases, which is beneficial to achieving China’s carbon emission reduction targets.

From the results in Table 1, we can see that the carbon tax recovery policy and technological progress are important conditions in achieving the triple dividend of the carbon tax. The carbon tax recycled to residents directly improves social welfare, and with the advancement of clean power technology, social welfare is further improved. While carbon tax recovery to companies initially hurts the welfare of residents, social welfare improves when clean electricity technology advances to a certain level. Finally, at the 10% level of clean electricity technology, carbon tax recycling to corporates realizes the triple dividend of the carbon tax. Compared to the baseline scenario, GDP rises, carbon reduction intensity decreases significantly, and social welfare improves.

### 4.2. The Impact of Policy Scenarios on Carbon Dioxide Emissions

Based on the impact of carbon tax policy and clean power technology progress on CO_2_ emissions, we simulated the relationship between carbon tax recovery with clean power technology advancement and a carbon tax rate under the 5% emission reduction constraint, analyzing the emission reduction contribution of different fossil energy sources. We only selected the 5% clean power technology progress rate as a representative value. Table 2 displays the carbon tax rate and fossil energy emission reduction contribution under different policy simulation combinations.

Under specific CO_2_ emission reduction targets, the carbon tax rate was determined. Regardless of the carbon tax recovery policy, with the technological advancement of the clean power sector, the carbon tax rate declines. This results from technological advances in clean power, where more fossil energy is replaced by clean power, with less fossil energy consumption and lower carbon emissions per unit. As a result, the carbon tax rate declines under a set carbon reduction target. We found that technological advances in appropriate clean electricity lower the carbon tax rate, which is beneficial for promoting carbon tax policies. 

As seen in Table 2, the ad valorem tax rate on coal is relatively high. This is because coal has a higher carbon content and a higher carbon dioxide emission coefficient than oil–gas. Coal is more impacted by carbon tax policies and bears more taxes. Similarly, with the advancement of clean power technology, ad valorem tax rates for coal and oil–gas fall.

In terms of emission reduction contribution, it is not difficult to find that coal emission reduction’s contribution is enormous, while the oil–gas emission reduction contribution is relatively low. Being a major source of CO_2_ emissions in China, coal has a high carbon emission coefficient, which is the key to carbon emission reduction. Therefore, the use of coal resources should be reduced. Additionally, coal-to-electricity technology should be promoted, with relatively clean energy sources as an alternative. 

### 4.3. The Impact of Policy Scenarios on Electricity Energy

Refining the power sector helped us study the power energy changes in detail during the simulation period. In this regard, the focus was to analyze the impact of carbon tax recovery policies on power consumption under the progress of clean power technology and the impact of departmental thermal power consumption; thus, we demonstrated the transformation of the power structure further and analyzed the effect of the policy.

#### 4.3.1. Electricity Consumption

**Figure 2:**
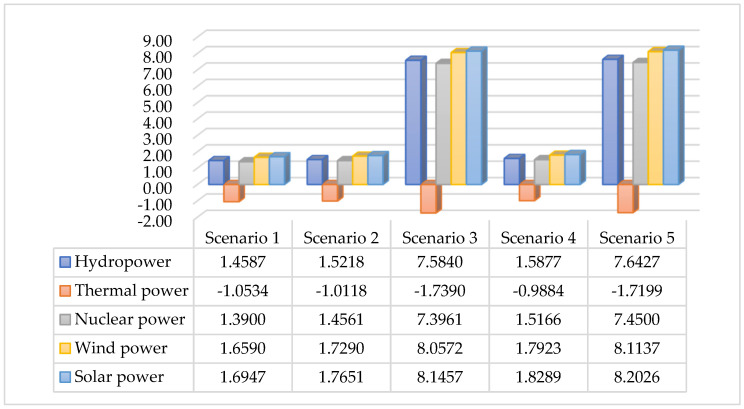
Percentage changes in electricity consumption (2017).

The collection of carbon tax policy affects the price of fossil energy and increases the production cost of enterprises. In this regard, the consumption demand of various energy sources will also change. In addition, technological progress in the clean power sector has a specific impact on various energy sources, especially electric energy. Selecting the 5% clean electricity technology progress rate as a representative value, we considered the changes in electricity consumption under carbon tax policies and different carbon tax recovery policies, as shown in Figure 2.

After implementing the carbon tax policy, coal and oil–natural gas were directly impacted. Corporates continue to seek alternative energy sources. Electricity, as an alternative energy source, is also indirectly affected. As a secondary energy source, thermal power indirectly emits carbon dioxide, and the levy of a carbon tax reduces the energy consumption of thermal power. Hydropower, nuclear power, wind power, and solar power generation, on the one hand, as clean energy, can replace fossil energy and thermal power and reduce carbon dioxide emissions. As a result, the consumption of clean power energy increases. On the other hand, as the rate of technological progress in the clean power sector increases, the clean power sector has increased production to replace thermal power generation. Thermal power consumption continues to decline, while clean power energy consumption increases significantly. The progress of clean power technology has a greater impact on the power industry. At the same time, the above results show that different power sectors have different responses to the impact of carbon taxes and technological progress, confirming the importance of subdividing the power sector.

#### 4.3.2. Sectoral Thermal Power Consumption

In China, coal is the primary raw material for thermal power generation. We focused on selecting thermal power sources with higher carbon dioxide emissions, analyzing the impact of carbon tax recovery policies and clean power technology progress on thermal power consumption in various sectors.

**Figure 3:**
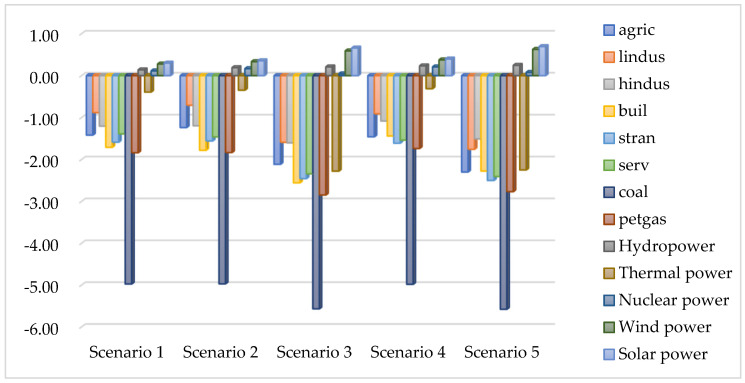
Percentage changes in sectoral thermal power consumption (2017).

Undoubtedly, from Figure 3, it can be seen that the consumption of thermal power in the clean power sector has increased, while the consumption of thermal power in other sectors has decreased. Among them, the coal sector has the largest decline in thermal power consumption, followed by oil–gas and construction industries. This is because the levy of a carbon tax increases the price of fossil energy and indirectly increases the cost of thermal power generation. The carbon tax has a relatively small impact on clean power, and China’s clean power accounts for a relatively small proportion. Enterprises’ demand for clean power increases, the clean power sector continues to grow, and the consumption of thermal power in the clean power sector increases. This change is noticeable, especially after the advancement of clean power technology, because the clean power sector develops more rapidly.

#### 4.3.3. Electricity Structure

The levy of a carbon tax increases the price of fossil energy, while the output of fossil energy and thermal power energy declines. Coupled with the progress of clean power technology, the output of clean power energy increases. In this regard, the power structure has been improved continuously.

**Figure 4:**
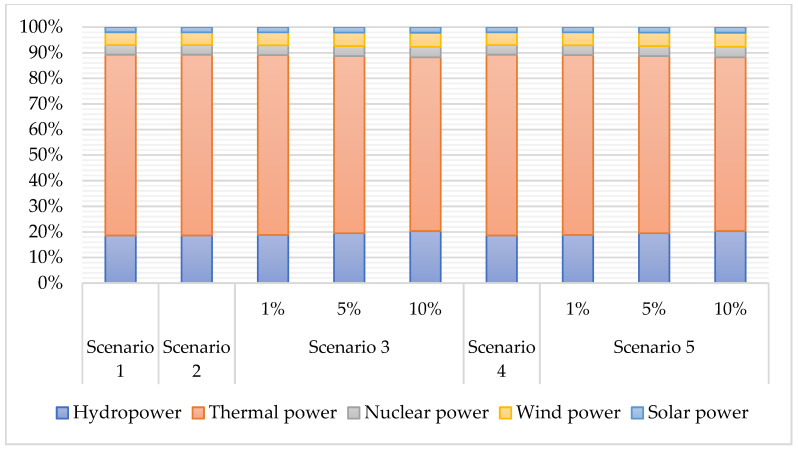
Changes in the power structure (2017).

As shown in Figure 4, thermal power is the main source of power generation. Progress in clean power technology significantly changes the power structure. With technological progress, the production efficiency of the clean power sector increases, and the output increases. In contrast, thermal power production declines. Therefore, the proportion of thermal power declines. The advancement of clean power technology is significant for changing the power structure and reducing carbon emissions. The proportion of thermal power is still large. Thus, reasonable measures need to be taken to adjust the power structure to reduce the proportion of thermal power continuously.

## 5. Conclusions and Policy Suggestions

This paper constructed a computable general equilibrium model of the power sector segmentation, taking into account the two tools for reducing carbon dioxide emissions, technological progress, and carbon tax, studying the economic–environment–energy effects of the carbon tax recovery policy with clean power technology progress. Through the above research, we have drawn several conclusions. First, a carbon tax recovery policy supplemented by technological progress in the clean power sector can promote economic growth, improve social welfare, and reduce the intensity of carbon dioxide emissions, realizing the triple dividend of the carbon tax. Carbon tax alone can reduce carbon emissions and adjust the energy structure, but the carbon tax policy will adversely affect economic development and social welfare [25,26,27]. A carbon tax recovery policy can remedy this problem. At the same time, the introduction of clean power technology advances make up for the negative impact of economic growth and social welfare losses, promoting the sustainable growth of the green economy. Second, advances in clean power technology promote the transformation of the power structure. The advancement of clean power technology drives the production of the clean power sector and replaces thermal power generation. Without difficulty, we found that the proportion of thermal power production declines, while the proportion of other clean power increases, which is conducive to promoting carbon emission reduction. Lastly, increasing the use of clean energy is the key to reducing carbon emissions. By analyzing the contribution of carbon dioxide emission reduction, we found that the contribution of coal emission reduction is relatively significant, and the ad valorem tax rate is high. The above is because coal has a higher carbon content and emits more carbon dioxide.

Based on the conclusions of the above research, we put forward the following policy recommendations for the future development of China’s economy. Firstly, we should pay attention to the role of carbon tax policy and carbon tax recycling. As an essential environmental fiscal policy, the carbon tax is an important measure needed to achieve carbon peak and carbon-neutral goals. However, China has not yet implemented a carbon tax policy and is still in the exploratory stage. The carbon tax policy can effectively supplement China’s existing ETS [57]. On this basis, we should adopt reasonable carbon tax recovery methods to reverse the adverse effects of carbon tax policies continuously. Secondly, we need to continue to accelerate technological progress in the clean power sector and deepen the technical research and development of China’s clean power sector and accelerate the transformation of electricity, that is, to promote the transformation of electricity from high-carbon to low-carbon sources, in a fossil-energy-based to clean-energy-based transformation. The substitution of thermal power and clean power is worthy of attention. We should vigorously develop clean power and reduce carbon dioxide emissions. Finally, we must promote clean coal and coal-to-electricity policies and accelerate the implementation of energy decarbonization policies. Coal significantly contributes to carbon emissions; therefore, it is essential that we accelerate and promote the transition from coal to electricity. Additionally, the state should increase the promotion of alternative renewable fuels [58].

This article provides a scientific basis for realizing carbon emission reduction targets and provides a particular reference for policymakers weighing economic development, environmental quality, and social welfare goals. However, there is still room for exploration in this article. First, a dynamic CGE model should be introduced to further compare the long and short-term effects of a carbon tax recovery policy along with the development of clean power technology on the power industry. Second, China’s economic system is relatively complex. To avoid one-sided research, more policy tools should be taken into consideration while exploring a more effective combination of carbon emission reduction and stable economic growth policies, contributing to the sustainable development of China’s green economy.

## Figures and Tables

**Table 1:** The impact of macroeconomic variables.

Scenarios	RTP	GDP (%)	RGDP (%)	TCOEI (%)	EV	YTH (%)	YTE (%)
Scenario 1	0%	−0.0377	−0.0493	−4.9641	−424.3465	0.0162	−0.0753
Scenario 2	0%	−0.0500	−0.0505	−4.9525	851.6128	−0.0144	−0.1022
Scenario 3	1%	−0.0457	−0.0334	−4.9566	865.6442	−0.0126	−0.0889
	5%	−0.0289	0.0335	−4.9725	922.0437	−0.0052	−0.0371
	10%	−0.0093	0.1144	−4.9912	993.0338	0.0034	0.0240
Scenario 4	0%	−0.0303	−0.0441	−4.9712	−530.8357	−0.0103	−0.0730
Scenario 5	1%	−0.0266	−0.0273	−4.9747	−470.2937	−0.0086	−0.0606
	5%	−0.0125	0.0389	−4.9881	−232.3547	−0.0018	−0.0127
	10%	0.0041	0.1189	−5.0039	55.9304	0.0062	0.0439

Note: RTP stands for the rate of technological progress for the clean power sectors; GDP stands for nominal GDP; RGDP stands for real GDP; TCOEI stands for total carbon dioxide emission intensity; EV stands for social welfare; YTH stands for the resident income; YTE stands for the corporate income.

**Table 2:** The carbon tax rate and contribution of fossil energy emission reduction.

Scenarios		1	2	3	4	5
Emission reduction contribution (%)	Coal	97.5820	97.6405	96.8750	98.1246	97.2784
	Oil–gas	2.4180	2.3595	3.1250	1.8754	2.7216
Ad valorem tax rate	Coal	0.0505	0.0508	0.0435	0.0517	0.0443
	Oil–gas	0.0129	0.0130	0.0111	0.0133	0.0113
Carbon tax rate (CNY/ton)		15.3563	15.4551	13.2062	15.7485	13.4477

## Data Availability

China Financial Yearbook (https://www.epsnet.com.cn/, (accessed on 23 January 2022)), China Statistical Yearbook (http://www.stats.gov.cn/tjsj/ndsj/, (accessed on 23 January 2022)), China Electricity Yearbook (https://navi.cnki.net/knavi/yearbooks/, (accessed on 23 January 2022)), China Industrial Statistics Yearbook (https://data.cnki.net/Yearbook/, (accessed on 23 January 2022)), China Input-Output Tables (http://data.stats.gov.cn/, (accessed on 23 January 2022)), International Energy Statistics (https://www.eia.gov/, (accessed on 23 January 2022)).
